# Higher CD4^+^CD40^+^ T cells (Th40 cells) associate with systemic lupus erythematosus activity

**DOI:** 10.1038/s41598-023-37749-y

**Published:** 2023-07-03

**Authors:** Lihua Zhu, Guangmei Song, Xiaohui Chen, Yue Zhang, Yanjie Cui, Jie Qiao, Xinran Huang, Xueqin Li, Xiaoen Liu, Xiangbo Zeng, Yangqiu Li, Liang Wang, Bo Li

**Affiliations:** 1 Department of Rheumatology and Immunology, First Affiliated Hospital, Jinan University, Guangzhou, 510632 China; 2 Institute of Hematology, School of Medicine, Jinan University, Guangzhou, 510632 China; 3 Department of Oncology, First Affiliated Hospital, Jinan University, Guangzhou, 510632 China

**Keywords:** Systemic lupus erythematosus, T cells, CD4-positive T cells

## Abstract

The aim of this study was to investigate the characteristics of CD4^+^CD40^+^ T cells (Th40 cells) in Chinese systemic lupus erythematosus (SLE) patients. Flow cytometry was used to identify the percentage of Th40 cells in peripheral blood from 24 SLE patients and 24 healthy individuals and the level of IL-2, IL-4, IL-6, IL-10, IFN-r, and TNF-α in serum (22 cases) from the SLE patients. Systemic Lupus Erythematosus Disease Activity Index 2000 (SLEDAI-2000) was used to assess the SLE disease active state. The percentage of Th40 cells in T cells from SLE patients (19.37 ± 17.43) (%) was significantly higher than that from healthy individuals (4.52 ± 3.16) (%) (*P* < 0.001). The percentage of Th40 cells was also positively associated with SLEDAI-2000 (*P* = 0.001) and negatively associated with complement C3 (*P* = 0.007). The Th40 cell percentage was different in SLE patients with different organs involved. The Th40 cell percentage in SLE patients with lupus serositis (29.29 ± 22.19) was significantly higher than that in patients without serositis (13.41 ± 10.79; *P* = 0.040), and the percentage in SLE patients with lupus pneumonia involvement (29.11 ± 11.88) was significantly higher than that in patients without lupus pneumonia (16.80 ± 17.99; *P* = 0.043). After 4 weeks treatment, the Th40 cell percentage decreased significantly (*P* = 0.005). However, Th40 cell expression was not related to cytokines (IL-2, IL-4, IL-6, IL-10, IFN-r, and TNF-α; *P* > 0.05). A significantly higher percentage of Th40 cells was found in SLE patients, and the Th40 cell percentage was associated with SLE activity. Thus, Th40 cells may be used as a predictor for SLE disease activity and severity and therapeutic efficacy.

## Introduction

Systemic lupus erythematosus (SLE) is a representative systemic autoimmune disease characterized by loss of tolerance to autoantigens, a variety of immune abnormalities, and a high titer of autoantibodies directed against nuclear components. This disease is highly heterogeneous, and different patients may have distinct symptoms and clinical characteristics. The pathogenesis of SLE is complex, and it is thought that genetic susceptibilities and environmental factors play a key role in the development of SLE^1–4^. Many T cell and B-cell abnormalities have been described in SLE, and this disease has been described to be a T cell-dependent autoimmune disease where CD4^+^ T cells play an important pathogenetic role^5,6^.

CD4^+^CD40^+^ T cells (Th40 cells) are a CD4^+^ T cell subset that expresses CD40, which is pathogenic in type I diabetes (T1D)^7–12^. Th40 cells, like regulatory T cells (Tregs), are developed in the thymus. These cells concomitantly secrete IFN-r and IL-17^13^. When a cognate antigen for a specific T cell receptor (TCR) is present, many more Th40 cells develop. These cells were able to ablate CTLA-4 expression and indirectly impact tolerance in a neo-self antigen disease model^14^. While Th40 cells are present in non-autoimmune strains (up to 25%), they expand to approximately 60% of the CD4^+^ T cell compartment in non-obese diabetic (NOD) mice, a model for type 1 diabetes (T1D) and, coincidentally, a model for relapsing–remitting experimental autoimmune encephalomyelitis (EAE)^13^. Primary, peripheral Th40 cells successfully transfer T1D without additional requirements^8,10,11^. Th40 cells also occur at significantly increased percentages in human T1D subjects compared with those with type 2 diabetes (T2D) and healthy controls^12^.

There are a number of studies of the susceptibility of Th40 cells in T1D and other autoimmune diseases. However, little is known about the association between Th40 cells and SLE. The aim of this study was to investigate the expression of Th40 cells in Chinese SLE patients.

## Results

### Higher percentage of Th40 cells in patients with SLE

**Figure 1:**
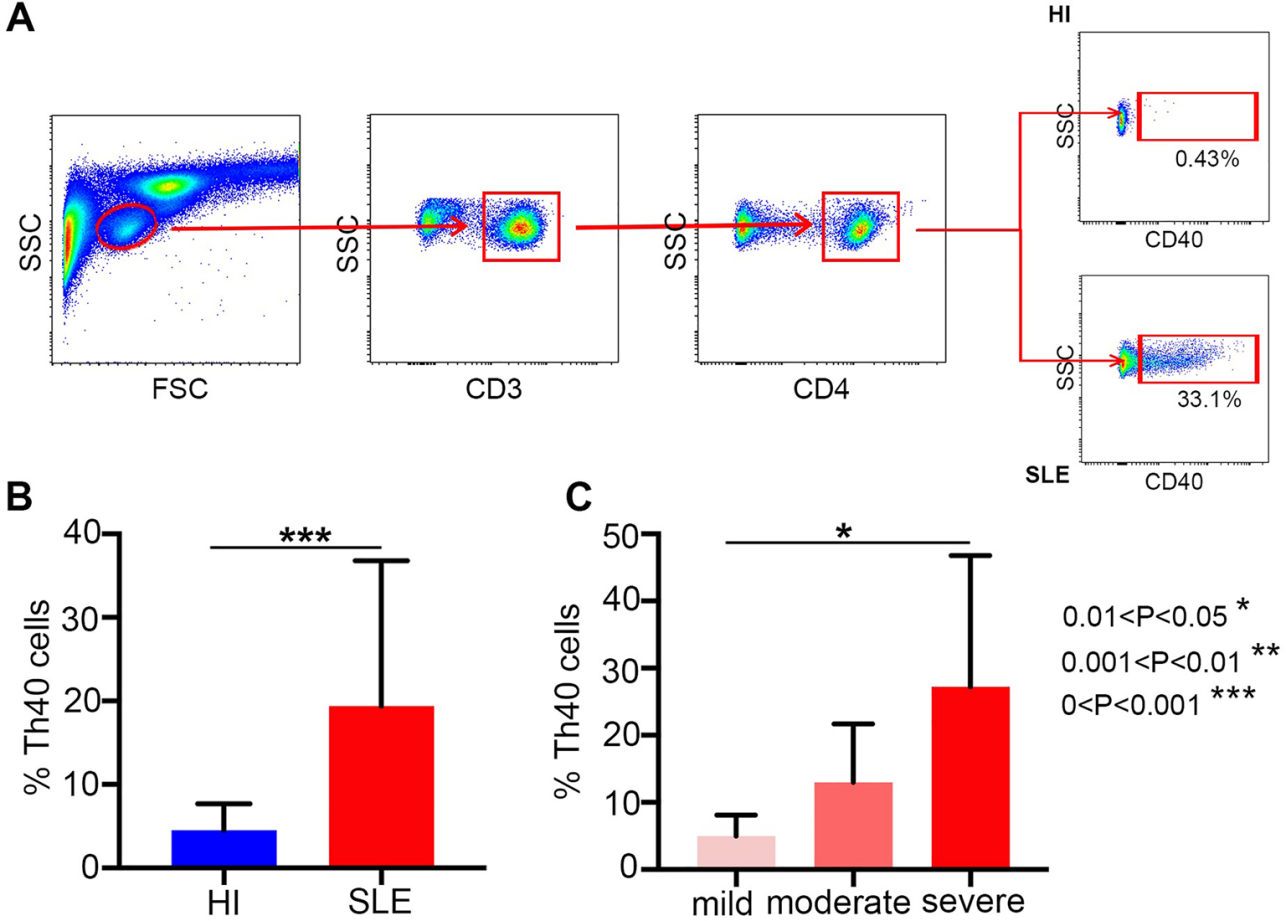
(**A**) The panels in this section show the gating strategy employed for the analysis of Th40 cells. Peripheral venous blood derived leukocytes were stained with different fluorescent antibodies and after lysis of red blood cells, the remaining cells were gated on living lymphocytes, gated on CD3^+^ T cells, and then gated on CD3^+^CD4^+ ^T cells, and then further gated on Th40 cells; (**B**) a higher percentage of Th40 cells was observed in SLE patients than in HI (healthy individuals); (**C**) a higher percentage of Th40 cells was observed in the severe activity group than in the mild activity group (*P < 0.05, **P < 0.01, ***P < 0.01).

We analyzed the percentage of Th40 cells in peripheral blood samples from 24 SLE patients and 24 healthy individuals by flow cytometry (Fig. 1A). All SLE patients were from first diagnosis without any drug therapy and in the disease active phase (SLEDAI-2000 ≥ 4). A significantly higher percentage of Th40 cells in T cells from SLE patients (19.37 ± 17.43) was found compared with that from healthy individuals (4.52 ± 3.16; *P* < 0.001; Fig. 1B).

### Higher percentage of Th40 cells associated with SLEDAI-2000 and complement C3

**Figure 2:**
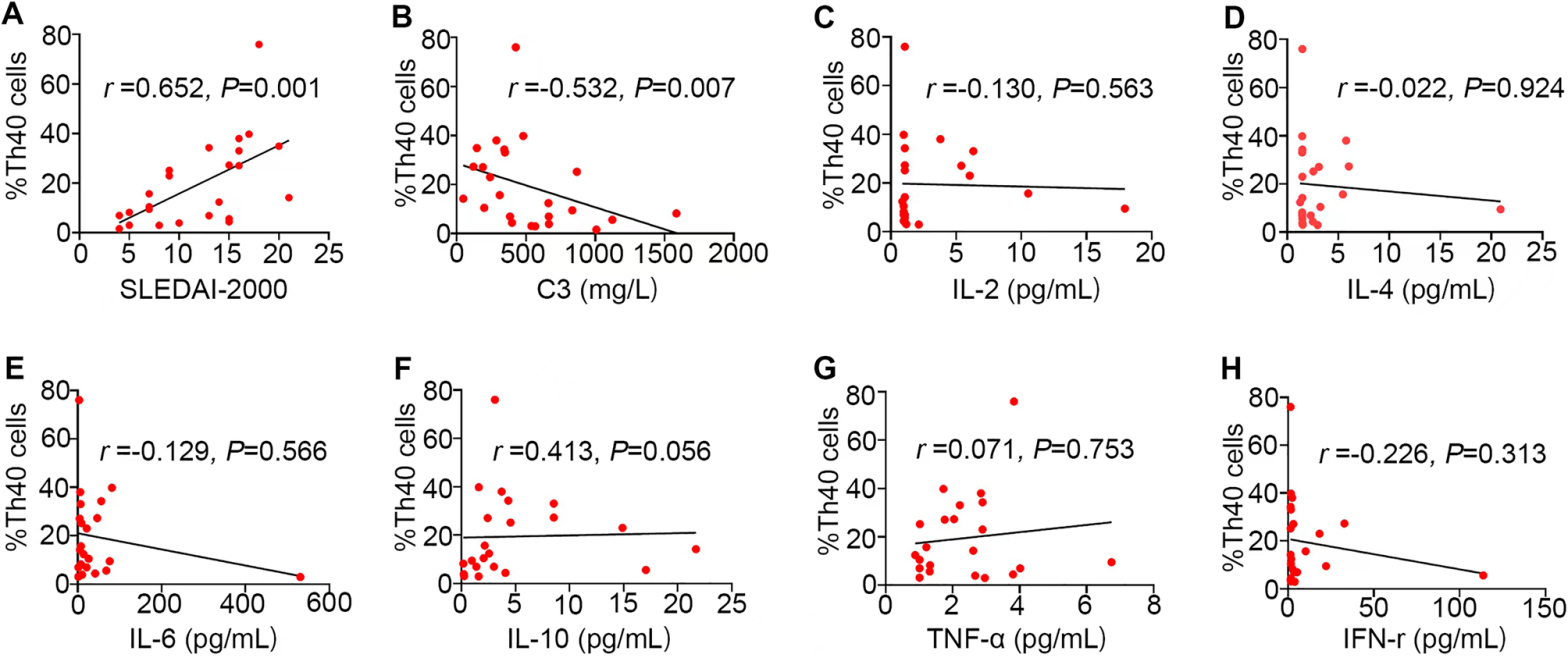
(**A**) Higher percentage of Th40 cells was positively associated with SLEDAI-2000; (**B**) higher percentage of Th40 cells was negatively associated with complement C3; (**C**–**H**) no statistical significance between Th40 cells and these cytokines.

We also analyzed the relationship between the percentage of Th40 cells and SLEDAI-2000. Patients with SLE were divided as mild activity (≤ 6), moderate activity (7–12), and severe activity (≥ 12) by SLEDAI-2000. The percentage of Th40 cells in 24 SLE untreated patients at initial diagnosis (activity disease) was positively associated with SLEDAI-2000 (r = 0.652, *P* = 0.001; Fig. 2A). The percentage of Th40 cells was (4.98 ± 3.14) in the mild activity group (n = 5), (12.97 ± 8.73) in the moderate activity group (n = 7), and (19.37 ± 17.43) in the severe activity group (n = 12). The percentage of Th40 cells was higher in the severe activity group than in the mild active group (*P* = 0.021; Fig. 1C).

We analyzed the relationship between the percentage of Th40 cells and IgG, IgA, IgM, complement C3 and C4, ESR and Hs-CRP (Supplementary Table 1). We found that the Th40 cell percentage was negatively associated with complement C3 (r = − 0.532, *P* = 0.007; Fig. 2B). There was no statistical significance (*P* > 0.05) found when analyzing IgG, IgA, IgM and C4. Further research involving more samples is needed to determine representative results. We also analyzed the relationship between the percentage of Th40 cells and the levels of serum cytokines (IL-2, IL-4, IL-6, IL-10, IFN-r, and TNF-α) in SLE (Supplementary Table 1). However, there was no statistical significance (*P* > 0.05) found between Th40 cells and these cytokines (Fig. 2C–H). Further research involving more samples is needed to determine representative results as well.

### The percentage of Th40 cells was different in SLE with different organs involved

We also analyzed the percentage of Th40 cells in 24 SLE patients with different organs involved including from patients with lupus nephritis, lupus blood system damage, lupus serositis, lupus pneumonia, neuropsychiatric lupus, skin erythema, and arthritis (Supplementary Table 2). The Th40 cell percentage in SLE patients with lupus serositis (29.29 ± 22.19) (n = 9) was significantly higher than that in individuals without serositis (13.41 ± 10.79; *P* = 0.040) (n = 15). The Th40 cell percentage in SLE patients with lupus pneumonia involvement (29.11 ± 11.88) (n = 5) was significantly higher than that in patients without lupus pneumonia (16.80 ± 17.99; *P* = 0.043) (n = 19). There was no statistically significant difference (*P* > 0.05) in SLE with other organs involved. Further research involving more samples and different organs is needed to determine representative results.

### The percentage of Th40 cells decreased after treatment

**Figure 3:**
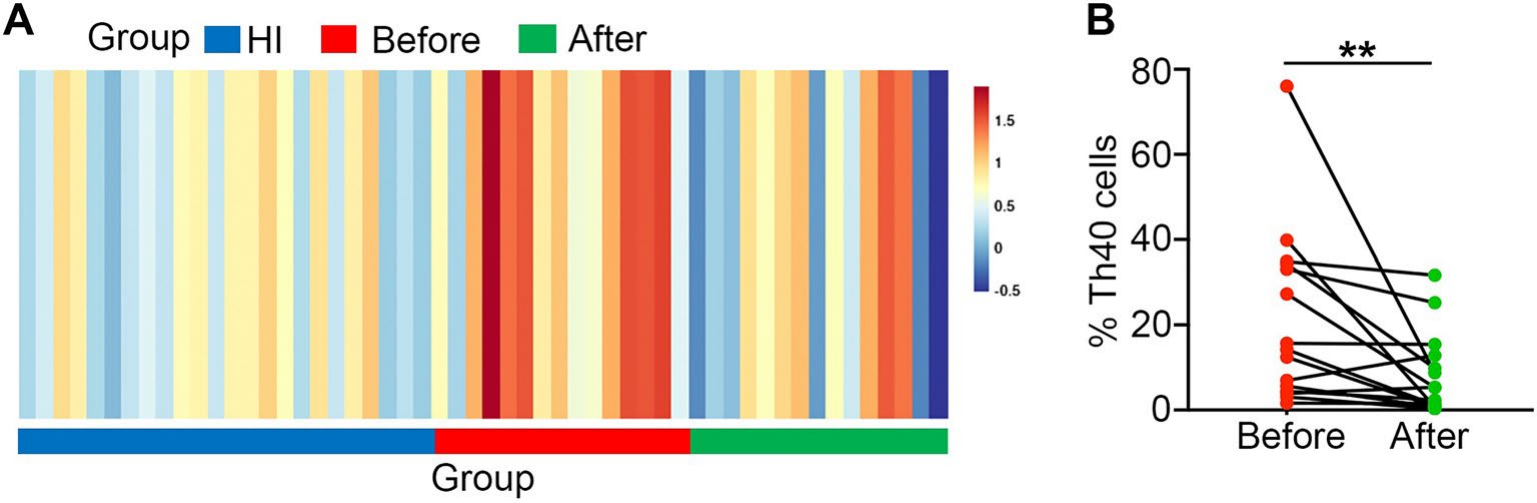
(**A**) OmicStudio 3.0 software was used to create Heatmap representing the percentage of Th40 cells in SLE patients before and after treatment and in HI (healthy individuals). The percentage of Th40 cells was higher in SLE patients before treatment than after treatment and HI; (**B**) the percentage of Th40 cells decreased after treatment in SLE (*P* = 0.005) (**P < 0.01).

We analyzed the percentage of Th40 cells in 15 untreated SLE patients at initial diagnosis (activity disease; 20.89 ± 20.32) and after 4 weeks of treatment with the same drugs (glucocorticoid, hydroxychloroquine, and cyclophosphamide) (8.14 ± 9.59). We found that the percentage of Th40 cells decreased significantly (*P* = 0.005; Fig. 3). The Th40 cell percentage in 15 SLE patients before treatment (20.89 ± 20.32) was higher than that in 24 healthy individuals (4.52 ± 3.16; *P* = 0.001). Nevertheless, there was no significant difference in the Th40 cell percentage after 4 weeks of treatment between SLE patients and 24 healthy individuals (*P* = 0.977).

## Discussion

Studies have revealed that non-autoimmune human subjects maintain peripheral levels of Th40 cells at up to 30% of the CD4^+^ T cell compartment^10,14^. Similar to a T1D animal model, human subjects with T1D^10^ and multiple sclerosis (MS)^14^ have an expansion of the Th40 cells (up to 50% or more of the CD4^+^ T cell compartment) in peripheral blood when compared to controls. Subjects with T2D, a non-autoimmune, chronic disease, do not demonstrate this expansion^12,15,16^. Th40 cell expansion is more predictive of T1D than the HLA-DR haplotype, and these cells are highly responsive to T1D autoantigens^12^. The same is true of Th40 cells in MS^15^. Th40 cells are pathogenic in T1D, EAE, and MS but have not been evaluated in SLE.

In this study, we for the first time investigated the percentage of Th40 cells in Chinese SLE patients. We demonstrated that Th40 cells in T cells from SLE patients with disease activity are significantly higher than that in healthy individuals. The percentage of Th40 cells in this study was lower than in other studies^12,15^, which is mostly likely because of different races and species. We further investigated the role of Th40 cells in Chinese SLE patients. The results demonstrated that Th40 cells in T cells from the blood of SLE patients with disease activity was significantly higher than that in healthy individuals. The percentage of Th40 cells was positively associated with SLEDAI-2000, and this was much higher if an SLE patient had more than one organ involved. We found that higher SLEDAI-2000 scores led to higher percentages of Th40 cells. Thus, the Th40 cell percentage was relative to SLE disease activity. We also found that the percentage of Th40 cells was negatively associated with complement C3, and it is well known that complement C3 is negatively associated with SLE disease activity. This finding further confirms that the percentage of Th40 cells is positively associated with SLE disease activity. Therefore, Th40 can be used as an indicator to assess SLE disease activity. Moreover, we found that the Th40 cell percentage in SLE patients with lupus serositis or lupus pneumonia was significantly higher than that in patients without serositis or pneumonia. However, in this study, we could not test all types of SLE patients with different organs involved. Further research involving more samples and different organ involvement is needed to determine representative results. All SLE patients accepted glucocorticoid, hydroxychloroquine, and cyclophosphamide therapy. We found that the Th40 cell percentage from SLE patients at initial diagnosis decreased significantly after 4 weeks of treatment. SLEDAI-2000 decreased significantly after 4 weeks treatment as well, and complement C3 was significantly elevated. Th40 cells (%) decreased significantly when SLE patients reached remission or low disease activity after 1 month. Therefore, the percentage of Th40 cells can be used as an indicator to evaluate the efficacy of disease treatment. At present, there are few indicators available to evaluate the severity of SLE. This study further demonstrated that the Th40 cell percentage is associated with disease activity in SLE. It may be that the combination of the Th40 cell percentage, SLEDAI-2000, and complement C3 can help to predict SLE disease activity, distinguish its severity, and predict the efficacy of therapy. We analyzed the levels of the serum cytokines IL-2, IL-4, IL-6, IL-10, IFN-r, and TNF-α in SLE. IL-6 is thought to play an important role in the regulation of the human immune system and is considered to play an important role in autoimmune diseases^17^. However, the percentage of Th40 cells was not related to IL-6 and other cytokines. Further research involving more samples is needed to determine representative results. Th1/Th2 related cytokines include IL-2, IL-4, IL-6, IL-10, IFN-r, and TNF-α. However, the percentage of Th40 cells was not related to these cytokines. Further researches involving more samples, in-vitro cell culture experiments, animal experiments are needed to determine representative results.

In conclusion, the percentage of Th40 cells was significantly positively correlated with the SLEDAI-2000 and inversely correlated with C3 levels. Perhaps Th40 cells can be used as a predictor of SLE disease activity and severity and efficacy of therapy. However, further research involving more samples is needed. It will be important to further understand the events leading up to disease onset and to elucidate the contributions of Th40 cells.

## Materials and methods

### Patients and samples

This study included 24 cases with untreated SLE (3 males and 21 females, age: 20–56 (29.33 ± 10.6) years, and 24 healthy individuals (3 males and 21 females, age: 24–46 (33.96 ± 6.01) years, who served as controls (Table 1). All SLE patients were from first diagnosis without any drug therapy and assessed for clinical disease activity by a trained rheumatologist using SLEDAI-2000. The exclusion criteria for the SLE sample was as follows: (1) combined with other autoimmune diseases. (2) Combined with other internal medicine diseases (type 1 diabetes, type 2 diabetes, hypertension, chronic kidney disease and so on). (3) Patients with severe infections. (4) Patients with malignant tumors. The healthy individuals were in a healthy status without any cancer, type 1 diabetes, type 2 diabetes, hypertension, or autoimmune inflammatory disease. Neither SLE patients nor healthy individuals smoked. All procedures were conducted according to the guidelines of the Medical Ethics Committee of the Health Bureau of the Guangdong Province in China.

**Table 1:** Clinical characteristics of the SLE patients and healthy controls.

Characteristics	SLE patients Mean ± SD/n (%)	Healthy controls Mean ± SD/n (%)
Female, n (%)	21 (87.5)	21 (87.5)
Male, n (%)	3 (12.5)	3 (12.5)
Age, years	29.33 ± 10.68	33.96 ± 6.01
ANA (+), n (%)	24 (100.00)	0
Anti-dsDNA (+), n (%)	16 (66.67)	0
Anti-Sm (+), n (%)	10 (41.67)	0

### Flow cytometry

Flow cytometry was used to identify Th40 cells in peripheral blood (24 cases) from SLE patients and 24 healthy individuals.

#### Antibodies and reagents

For flow cytometry, CD3-FITC (HIT3a) and CD40-PE/Cyanine·7 (5C3) were obtained from BioLegend (San Diego, USA); CD4-APC-H7 (RPA-T4) was obtained from BD Pharmingen (San Diego, USA); Red blood cell lysis buffer and phosphate-buffered saline (PBS) were obtained from BD Biosciences (San Jose, USA).

#### Flow cytometry analysis

For all cytometric analyses, at least 1 × 10^6^ cells were obtained by red blood cell lysis of blood from patients with SLE or healthy individuals followed by analysis using a BD FACSVerse multicolor flow cytometer (BD Biosciences). Cells were incubated with specific antibodies directed against surface markers including anti-CD3, anti-CD4, and anti-CD40. Then, the cells were washed and resuspended to prepare for detecting the CD40 percentage in the CD4^+^ T cell subsets (Fig. 1A).

Flow cytometry (Guangzhou KingMed Diagnostics Group Co., Ltd.) was also used to analyze the levels of serum cytokines IL-2, IL-4, IL-6, IL-10, IFN-r, and TNF-α in serum (22 cases) from SLE patients.

### Statistical analysis

All statistical analyses were performed with SPSS 23.0 software. And a P value of less than 0.05 was considered statistically significant. Student’s t-test was used to compare the differences in continuous variables with normal distributions, and the Mann–Whitney U test was used for continuous variables with non-normal distributions between two groups. The χ^2^ test and Fisher’s exact test were used to compare the differences in categorical variables. The correlation analysis was performed using Pearson's or Spearman's correlation analysis. Data are expressed as mean ± SD unless otherwise specified.

### Ethical approval

This study was approved by the Ethics Committee of First Affiliated Hospital, Jinan University. The methods were carried out in accordance with the principles stated in the Declaration of Helsinki. Informed consent was obtained from each patient.

## Supplementary Information

Supplementary Tables.

## Data Availability

The datasets used and analysed during the current study available from the corresponding author on reasonable request.
