# Supplementary material for: Higher CD4+CD40+ T cells (Th40 cells) associate with systemic lupus erythematosus activity
Source: Sci Rep. 2023 Jul 3;13:10702. doi: 10.1038/s41598-023-37749-y (PMC10317960; doi:10.1038/s41598-023-37749-y)
Supplement: Supplementary file 1 — Supplementary Tables. [file 41598_2023_37749_MOESM1_ESM.doc]

Supplementary Table 1 Correlation analysis of Th40% and clinical data

|  | Nnumber | SLE patients  Mean ± SD | Th40% | |
| --- | --- | --- | --- | --- |
|  |  |  | r | *P* |
| SLEDAI-2000 | 24 | 11.83±5.21 | 0.652 | **0.001** |
| IgA (g/L) | 24 | 271±1.19 | 0.105 | 0.626 |
| IgG (g/L) | 24 | 20.34±7.44 | 0.086 | 0.689 |
| IgM (g/L) | 24 | 1.05±0.59 | 0.037 | 0.865 |
| C3 (mg/L) | 24 | 518.41±363.33 | -0.532 | **0.007** |
| C4 (mg/L) | 24 | 105.82±95.72 | -0.245 | 0.248 |
| ESR (mm/h) | 24 | 60.75±37.69 | 0.416 | 0.068 |
| Hs-CRP (mg/L) | 24 | 18.04±25.32 | 0.041 | 0.865 |
| IL-2 (pg/mL) | 22 | 3.08±4.17 | -0.130 | 0.563 |
| IL-4 (pg/mL) | 22 | 3.28±4.20 | -0.022 | 0.924 |
| IL-6 (pg/mL) | 22 | 47.36±111.03 | -0.129 | 0.566 |
| IL-10 (pg/mL) | 22 | 4.95±5.01 | 0.413 | 0.056 |
| IFN-r (pg/mL) | 22 | 10.68±24.42 | -0.226 | 0.313 |
| TNF-α (pg/mL) | 22 | 2.35±1.40 | 0.071 | 0.753 |

**Supplementary Table 2 Th40% was different in SLE patients with different organs involved**

| Organs | organ-involved  (%) | non-organ-involved  (%) | *P* value |
| --- | --- | --- | --- |
|
| lupus nephritis | 26.03±20.67 | 11.49±7.76 | 0.068 |
| lupus blood system damage | 20.52±17.76 | 6.70±3.92 | 0.251 |
| lupus serositis | 29.29±22.19 | 13.41±10.79 | **0.040** |
| lupus pneumonia | 29.11±11.88 | 16.80±17.99 | **0.043** |
| neuropsychiatric lupus | 9.93±6.04 | 20.22±17.93 | 0.436 |
| skin erythema | 7.91±8.90 | 20.41±17.76 | 0.343 |
| arthritis | 14.25±12.19 | 21.47±19.10 | 0.357 |
